# Potential determinants of parental hesitancy to vaccinate their children against COVID-19 infection: a cross-sectional investigation

**DOI:** 10.1038/s41598-023-47863-6

**Published:** 2023-12-13

**Authors:** Shazia Rehman, Nadia Rehman, Zexuan Li, Yan Zhang

**Affiliations:** 1 Department of Psychiatry, National Clinical Research Center for Mental Disorders, National Center for Mental Disorders, The Second Xiangya Hospital of Central South University, Changsha, 410011 Hunan China; 2 China National Technology Institute on Mental Disorders, Hunan Technology Institute of Psychiatry, Hunan Key Laboratory of Psychiatry and Mental Health, Hunan Medical Center for Mental Health, Mental Health Institute of Central South University, Changsha, 410011 Hunan China; 3 Department of Mathematics, COMSATS University, Wah Campus, Islamabad, Pakistan

**Keywords:** Diseases, Health care

## Abstract

Coronavirus disease 2019 (COVID-19) vaccination hesitancy has emerged as a substantial concern among the adult population globally. However, limited evidence is available about parental hesitancy to vaccinate their children against COVID-19 within the Pakistani context. Thus, the present investigation aimed to assess parental attitudes, perceptions, and willingness regarding vaccination hesitancy and associated predictors of getting their children vaccinated against COVID-19. We conducted a cross-sectional population-based, self-administered online questionnaire in Punjab, Pakistan, on randomly selected parents between October 2022 and February 2023. The data were collected based on socio-demographics, attitudes, perceptions, and willingness of parents regarding COVID-19 vaccine hesitancy for their children. Adjusted odds ratios with 95% confidence intervals were estimated to identify the predictors of vaccine hesitancy. The findings demonstrated that among 1,478 participants, a total of 40% believed that the COVID-19 vaccine may pose a greater risk to children than adults, while 38% exhibited no concerns. Around 13% of children were not vaccinated in our study sample. More than half expressed hesitancy toward vaccination, and only 35.25% were inclined to get their children vaccinated in our study sample. In addition, only 16% of the parents believed that the COVID-19 vaccination may cause an alteration in their children’s DNA. A similar proportion of parents were aware of the significance of getting their children vaccinated and expressed their willingness to vaccinate their children to prevent the COVID-19 infection. However, a higher odds ratio was observed in females with a higher educational background and those in the healthcare profession. In conclusion, healthcare awareness-supporting programs for educating parents should be designed and implemented. These insights might aid in the development of strategies to eradicate barriers in existing coronavirus vaccination programs and may vaccinate a larger child population to reduce the adverse consequences of the pandemic.

## Introduction

The World Health Organization (WHO) put out COVID-19 a worldwide outbreak on March 11, 2020, and it has since expanded to nearly every country in the globe, significantly affecting society and the economy. Noticeably, there were over 200 million confirmed diagnoses and over 5 million fatalities until 30 November 2021^1,2^. To prevent the infection from spreading, a variety of physical strides have been adopted, such as wearing a mask and social seclusion. For children, the principal strategy has been to close or reduce school hours^3^. Social isolation, unfortunately, adversely affects children's psychological health^4^. The most reliable way to dramatically lessen the effects of COVID-19 infection and let kids resume their usual lives is through vaccination^5,6^. Both adequate vaccine production and substantial levels of acceptability are necessary for an immunization procedure to be efficacious. Excitingly, since the virus's genetic composition was revealed in January 2020, more than 100 possible vaccine alternatives have been discovered, and so forth^7^. As per the statistical approaches, 60–72% of individuals need to be inoculated to develop protective immune responses for vaccines that are about 80% effective^8^. Given the emergence of new polymorphisms with the considerable transmission of disease, such as the recent discovery of the Omicron variant, higher vaccination adoption may be warranted^9^.

Children infected with COVID-19 showed mild clinical concerns and reduced morbidity and fatality rates than adults^10–13^, however, they are nonetheless at risk of infection and may function as carriers at school and home^14,15^. Furthermore, the ongoing evolution of COVID-19 variants may enhance the infection rate and pathogenicity in children, posing a challenge to current preventative and controlling approaches^16^. The vaccination of adults is an important aspect of generating herd immunity and enabling communities to expand^17^. Based on these considerations, vaccinating children against COVID-19 is a vital component of long-term pandemic control^18–20^. To combat the COVID-19 catastrophe, over 300 COVID-19 vaccinations have been produced globally^21^. Though COVID-19 has mutated into several strains, evidence has confirmed that the current COVID-19 vaccines maintained neutralizing concentration with modestly decreased or constant potency^16^. Furthermore, clinical experiments have demonstrated that many vaccines are secure and efficient in combating against COVID-19 infection in children^22–24^. Many nations have approved authorization for immunization of children aged below 18. Pakistan has approved the three COVID-19 vaccines (Pfizer, Sinopharm and Sinovac) for use in children aged between12 and 18.

Vaccine hesitancy, as characterized by the World Health Organization (WHO) as the inclination to delay or decline immunization despite the availability of vaccination services, presents a formidable impediment to the widespread acceptance and uptake of vaccines during the current COVID-19 vaccine development phase^25^.

Pakistan poses a significant challenge in convincing its populace to get vaccinated. Pakistan possesses relatively a weak healthcare system, primarily attributed to the challenges presented by its high population density. Consequently, the nation faces heightened vulnerability to swift transmissions of viral diseases. Hence, throughout the epidemic, the country is confronted with the secondary challenge of persuading individuals to avail themselves of vaccination, in addition to the primary concern of managing the pandemic itself. In the early phase of the outbreak, the Pakistani population exhibited hesitancy towards accepting the vaccine; however, as time progressed, the dispelling of fallacious beliefs associated with the vaccine has been substantiated by the tangible evidence of numerous lives preserved. The aforementioned circumstances have facilitated the endorsement of vaccinations within the country^26^. Asian countries exhibited a notable level of confidence in the government's proficiency in administering vaccines, with a majority over 80% of individuals expressing willingness to receive the vaccination^27^. In addition, a considerable proportion of the Pakistani population, about 71%, exhibits a voluntary inclination towards receiving vaccination against the virus^28^.

The hesitancy exhibited by parents in vaccinating their children against COVID-19 represents a significant impediment to childhood vaccination efforts. The present occurrence of this phenomenon no longer facilitates the attainment of herd immunity throughout the pandemic. Concurrent with the formulation of the COVID-19 immunization strategy for minors, it is imperative to comprehensively comprehend the factors underlying parental vaccination hesitancy and their stance on COVID-19 vaccination. Therefore, it is crucial to alleviate vaccine hesitancy towards COVID-19 by acquiring a comprehensive understanding of its determinants to mitigate the ongoing pandemic. There is a dearth of empirical evidence regarding this subject, and to the best of the author's knowledge, no studies have been published from the Punjab province concerning the age group of 0–18 years thus far. Therefore, the present survey investigation aims to explore and evaluate parental attitudes, perceptions, and willingness concerning vaccination hesitancy and associated predictors to get their children vaccinated for coronavirus infection and to formulate targeted interventional measures to foster a favorable perspective towards the COVID-19 vaccination.

## Methods

### Data source and study population

To achieve the research goals, a closed-ended questionnaire was delivered electronically to the parents residing in the Punjab province between October 2022 and February 2023. We adapted the questionnaire for our survey from the work of Shati et al. to collect the data^29^. To obtain data more conveniently, using Google-based forms, a web-based survey in both English and Urdu languages was created and sent to the study’s participants via e-mails and social platforms. In order to facilitate effortless comprehension of the questionnaire and prevent any discrepancy, the English questionnaire was transcribed to Urdu (the official language) by a professional. Pilot testing was undertaken among randomly selected parents within the selected region prior to administering the final version of the questionnaire. This preliminary evaluation aimed to assess the validity and pertinence of the instrument. The results of the pilot study are available in the supplementary file 2. Participants in the pilot study were not included in the final study sample and were conducted via a link through Google Forms.

### Survey questionnaire

There were three categories in the survey questionnaire. The first category was comprised of social-demographic particulars of the study participants, whereas the second category was comprised of the questions to measure the attitude and perspectives of the study participants towards the vaccination of coronavirus infection for their children. The final category included the queries evaluating the parents’ willingness towards the COVID-19 vaccination for their children.

### Inclusion/exclusion criteria

**Figure 1:**
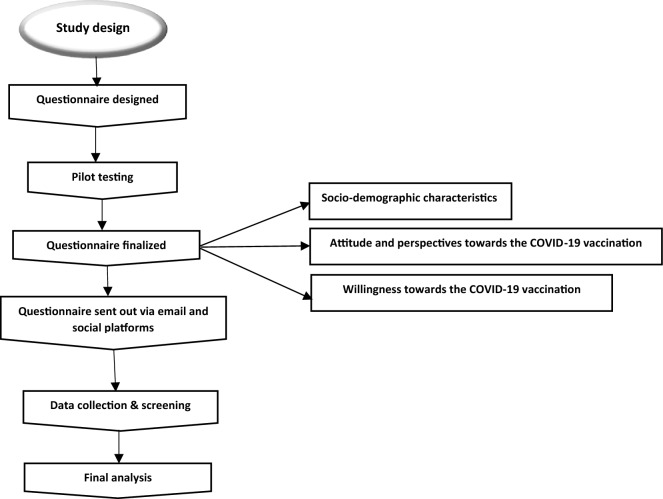
Study design.

The inclusion criteria were (i) male and female parents who were currently having children with ages between 0 and 18 years as well as those who were not having children, (ii) who could read and understand English/Urdu. The exclusion criteria were (i) parents with children above 18 years of old (ii) incomplete information provided (ii) participants who are unable to understand English/Urdu, and (iv) participants with any mental disability (Fig. 1).

### Sample size calculation

By supposing the maximum variation at 50% with $p=0.5$ and carrying level of confidence at $95\%$ with $\pm 5\%$ comparative specificity, the estimated sample size turned out to be 1537 using the formula $n={z}^{2}p(1-q)/{d}^{2} .$

Due to inaccurate or insufficient data, we eliminated 59 responses from the survey since they did not adequately reflect participants' preferences. Consequently, 1478 samples were taken for further analysis. Participants were not compensated with any privileges or finances as payment for answering the online survey. In the subsequent round, two biostatisticians reviewed and managed the collected data before entering it into MS EXCEL (v.2019) or for further analysis. Moreover, a STROBE checklist has been provided in the supplementary file 1.

### Statistical analysis

The collected data were entered and processed using MS Excel and SPSS (v27). Initially, Harman’s single-factor test was applied to assess the common method bias issue. Prior to the analysis, questionnaire items were tested for internal consistency using Cronbach’s alpha coefficient. The data were then analyzed for descriptive and inferential statistics. Descriptive statistics in the form of frequency and percentages were applied to the data along with a 95% confidence interval. For further analysis, an adjusted odds ratio with a 95% confidence interval was estimated to explore the determinants of parental hesitancy towards COVID-19 in getting their children vaccinated. A p-value less than 0.5 was considered to be statistically significant.

### Institutional review board statement

The study was conducted according to the guidelines of the Declaration of Helsinki, and approved by the Ethics Review Committee of the university (Ref.# PAF-IAST-2022/00237)).

### Informed consent statement

Informed consent was obtained from all subjects (parents) involved in the study. There was no direct involvement of children.

## Results

### Harman’s single-factor test for common method bias

The present study acknowledges the potential for biases inherent in self-reported data, and therefore, to ensure methodological rigor, Harman’s single-factor test was employed to examine the presence of common methodological biases before conducting data analysis. The findings of the study illustrated the presence of 2 factors exhibiting eigenvalues above 1, as shown in Table 1. The initial factor, which represented a substantial proportion of the variance at 31.56% (below the threshold of 40%), indicated the absence of any common biases in the study.

**Table 1:** Total variance explained.

Items	Initial eigenvalues			Extraction sums of squared loadings		
	Total	% of variance	Cumulative %	Total	% of variance	Cumulative %
1	4.103	31.56	31.56	4.103	31.56	31.56
2	2.99	23.00	54.56	2.99	23.00	54.56
3	0.848	14.22	61.08			
4	0.818	6.29	67.38			
5	0.717	5.52	72.89			
6	0.684	5.26	78.15			
7	0.612	4.71	82.86			
8	0.532	4.09	86.95			
9	0.489	3.76	90.71			
10	0.427	3.28	94.00			
11	0.339	2.61	96.61			
12	0.276	2.12	98.73			
13	0.165	1.27	100.00			

### Reliability analysis

We also examined the internal consistency (reliability) of the items on the survey questionnaire using Cronbach’s alpha coefficient. An alpha value exceeding 0.7 is considered acceptable, while > 0.8 is deemed good, and a value above 0.9 is considered excellent. Consequently, the outcomes demonstrated a good reliability of all the items in our study, as shown in Table 2.

**Table 2:** Reliability analysis.

Items on the questionnaire	α-coeff
Have you been hesitant to vaccinate your child?	0.86
Do you believe that your child should receive the COVID-19 vaccine?	0.87
Are you willing to vaccinate your child against COVID-19 infection?	0.87
Do you avoid giving your child the COVID-19 vaccine because you are afraid of the adverse effects?	0.85
Do you have fears regarding the potential impact of the COVID-19 vaccine on the pubertal development or fertility of your child?	0.86
Do you think it's a good idea to get the COVID-19 vaccine as soon as possible?	0.89
Do you have fears that the COVID-19 vaccine could disrupt your child's DNA?	0.88
What level of satisfaction do you have with the services offered at the vaccination centers?	0.85
Have you ever experienced any negative reactions from vaccinations?	0.88
Have you received the seasonal influenza vaccination the previous year?	0.86
Any of the following entities have been confirmed to have contracted the COVID-19 infection in a clinical setting?	0.89
Do you suspect that you may have had a Coronavirus infection or exposure without being tested?	0.88

α-coeff: Cronbach’s alpha coefficient.

### The univariate analysis of parents' socioeconomic demographic traits and their concern for the vaccination of their children

The univariate relationship between parents' socioeconomic demographic traits and their concern for the immunization of their children is summarized in Table 3. An aggregate of 1,478 samples was involved in the research investigation. 592 out of 1,478 parents believed that children may be at more risk from vaccination than adults. Female parents were more frequent than male parents among the 592 research participants. Around 21% of parents believe that vaccination is not necessarily riskier for children than it is for adults. Parental concerns regarding children's vaccinations were shown insignificant to approximately 38.84% (574 of 1478) study participants. More than 34% of parents in the 18 to 28 years-old age group believe that vaccinations may not be riskier for children than for grownups, while 44.03% disagreed. A significant proportion of participants with an educational background or bachelor's degree and those serving in the healthcare sector believed that vaccinations might not be more unsafe for children than grownups. Compared to parents who were childless, 43.25% of those who had children presumed that children might not be more at risk from immunizations than grownups. Parent's socio-economic demographic traits (such as sex, age, marital status, employment, occupation, having children, and the proportion of kids who are concerned about children’s vaccinations, were shown to differ statistically.

**Table 3:** The univariate analysis.

	Parents’s intent to COVID-19 vaccination				Significance
Socio-economic traits	Yes (n = 592), n (%)	No (n = 312), n (%)	Don’t know (n = 574), n (%)	Adj OR (95% CI)	
Gender					
Male (n = 560)	269 (48.04)	173 (30.89)	118 (21.07)	1.24 (0.68–2.26)	< 0.001
Female (n = 918)	323 (35.19)	139 (15.14)	456 (49.67)	1.65 (0.91–2.87)	
Age (years)					
18–28 (n = 511)	225 (44.03)	178 (34.83)	108 (21.14)	3.32 (2.78–4.52)	0.002
29–39 (n = 349)	159 (45.56)	88 (25.21)	102 (29.23)	2.76 (1.88–3.32)	
40–50 (n = 413)	151 (36.56)	88 (21.31)	174 (42.13)	3.08 (2.45–3.77)	
$\ge$ 51 (n = 205)	57 (27.80)	59 (28.78)	89 (43.41)	2.17 (1.84–2.99)	
Marital status					
Married (n = 1067)	331 (31.02)	333 (31.21)	403 (37.77)	1.58 (0.89–2.66)	< 0.001
Divorced (n = 195)	65 (33.33)	81 (41.54)	49 (25.13)	0.64 (0.32–1.21)	
Widowed (n = 216)	48 (22.22)	92 (42.59)	76 (35.19)	0.98 (0.28–1.39)	
Educational level					
Uneducated (n = 14)	5 (35.71)	6 (42.86)	3 (21.43)	0.34 (0.09–0.81)	< 0.001
Primary (n = 416)	128 (30.77)	120 (28.85)	168 (40.38)	2.01 (1.45–2.76)	
Bachelor (n = 901)	285 (31.63)	301 (33.41)	315 (34.96)	2.79 (1.63–3.42)	
Masters (n = 147)	32 (21.77)	67 (45.58)	48 (32.65)	1.54 (0.76–2.03)	
Occupation					
Medical (n = 237)	63 (26.58)	120 (50.63)	54 (22.78)	0.73 (0.12–1.17)	< 0.001
Non-medical (n = 1241)	412 (33.19)	321 (25.87)	508 (40.93)	1.15 (0.76–1.89)	
Employment					
Employed (n = 799)	243 (30.41)	251 (31.41)	305 (38.17)	2.87 (2.09–3.37)	0.002
Unemployed (n = 488)	181 (37.09)	150 (30.74)	157 (32.17)	2.11 (1.76–2.90)	
Retired (n = 191)	43 (22.51)	52 (27.23)	96 (50.26)	1.63 (1.11–2.23)	
Pre-existing comorbidity					
Yes (n = 163)	53 (32.52)	35 (21.47)	75 (46.01)	2.45 (1.69–3.17)	0.002
No (n = 1315)	426 (32.40)	441 (33.54)	448 (34.07)	3.13 (2.59–3.81)	
Having children?					
Yes (n = 1152)	329 (28.56)	302 (26.22)	521 (45.23)	2.64 (2.11–3.17)	< 0.001
No (n = 326)	90 (27.61)	141 (43.25)	95 (29.14)	2.05 (1.45–2.95)	
No. of children					
$\le 2$ (n = 857)	290 (31.94)	300 (33.04)	318 (35.02)	1.19 (0.89–1.97)	< 0.001
$>2$ (n = 621)	181 (31.75)	160 (28.07)	229 (40.03)	0.86 0.24–1.32)	

### The variation in the parental perspectives on the COVID-19 vaccination

The variation in parental perspectives on the COVID-19 vaccination can be seen in Table 4. Among 1478 study participants, 51.62% of individuals exhibited hesitancy towards vaccination, whereas approximately 35.25% promptly facilitated the vaccination of their children without any hesitation. On the contrary, only 13.13% of the research participants abstained from expressing any reservations about vaccination. A proportion of 60.42% of participants agreed that parents must vaccinate their children against COVID-19. Additionally, 61.77% of the participants showed their willingness to vaccinate their kids to protect them from contracting COVID-19. Moreover, 32.34% of parents reported having concerns about the negative effects of the vaccination that would prevent them from giving it to their children to protect them from COVID-19. Likewise, 28.00% of the participants expressed fear that the COVID-19 vaccine would influence their children's fertility or puberty; however, 35.25% of parents expressed complete disagreement with this fear. The survey investigation demonstrated that over 80% of participants suggested rushing to get the COVID-19 vaccination, while 62.25% of participants agreed that the services offered at the vaccination centers were excellent. Besides, 914 out of 1478 participants reported that their source of information about COVID-19 vaccinations was healthcare professionals, whereas 38.16% of the participants reported that their source of vaccination awareness was electronic media.

**Table 4:** Parental perspectives about COVID-19 vaccine.

	Frequency (%)	95% confidence interval
Have you been hesitant to vaccinate your child?		
Yes	521 (35.25%)	29.52–42.38
No	763 (51.62%)	42.78–65.34
Not received	194 (13.13%)	5.98–23.71
Do you believe that your child should receive the COVID-19 vaccine?		
Strongly agreed	366 (24.76%)	17.38–35.09
Agreed	527 (35.66%)	26.03–45.19
Neutral	398 (26.93%)	19.32–35.07
Do not agree	110 (7.44%)	1.19–14.93
Strongly disagree	64 (4.33%)	0.67–7.31
Do not know	13 (0.88%)	0.04–1.54
Are you willing to vaccinate your child against COVID-19 infection?		
Strongly agreed	406 (27.47%)	19.42–36.01
Agreed	507 (34.30%)	24.83–43.17
Neutral	301 (20.37%)	11.29–28.06
Do not agree	120 (8.12%)	2.10–12.37
Strongly disagree	23 (1.56%)	0.03–2.10
Do not know	121 (8.19%)	1.23–10.28
Do you avoid giving your child the COVID-19 vaccine because you are afraid of the adverse effects?		
Strongly agreed	297 (20.09%)	13.50–25.44
Agreed	366 (24.76%)	18.03–33.13
Neutral	242 (16.37%)	10.56–20.08
Do not agree	287 (19.42%)	13.16–27.05
Strongly disagree	191 (12.93%)	4.28–18.33
Do not know	95 (6.43%)	1.09–9.85
Do you have fears regarding the potential impact of the COVID-19 vaccine on the pubertal development or fertility of your child?		
Strongly agreed	101 (6.83%)	1.10–10.04
Agreed	313 (21.18%)	16.40–28.74
Neutral	543 (36.74%)	29.72–44.41
Do not agree	359 (24.29%)	18.62–32.19
Strongly disagree	162 (10.96%)	5.06–16.72
Do not know	0 (0.00%)	–
Do you think it's a good idea to get the COVID-19 vaccine as soon as possible?		
Yes	1157 (78.28%)	73.04–81.17
No	255 (15.22%)	11.32–18.63
Do not know	66 (4.47%)	1.56–8.93
Do you have fears that the COVID-19 vaccine could disrupt your child's DNA?		
Yes	237 (16.04%)	12.09–21.13
No	606 (41.00%)	36.54–48.19
Do not know	635 (42.96%)	38.02–49.92
What level of satisfaction do you have with the services offered at the vaccination centers?		
Excellent	920 (62.25%)	54.62–68.13
Very good	341 (23.07%)	17.16–27.16
Good	157 (10.62%)	6.71–16.54
Acceptable	51 (3.45%)	1.14–6.23
Not satisfied	9 (0.61%)	0.07–0.82
What sources do you use to get your knowledge regarding vaccines?		
Healthcare experts	914 (61.84%)	57.04–67.84
Electronic media	564 (38.16%)	30.19–44.38
Any other source	–	–

### The variation in parental experience with vaccination, immunity, and the COVID-19

The frequency and percentages regarding the parental experience of vaccinations and COVID-19 are displayed in Table 5. Among 1478 participants, approximately 19.49% reported experiencing adverse effects after vaccine acquisition. In addition to it, specifically 38.29% of the participants, availed themselves of the seasonal influenza vaccine during the preceding year. Contrarily, the remaining 61.71% of participants did not receive the seasonal influenza vaccination in the previous year. Further, laboratory screening revealed that about 20% of the participant's relatives, followed by the participants themselves (18.34%) and the participant’s friends (9.68%), were infected with COVID-19. The findings also revealed that a significant proportion of the participants (58.66%), expressed a lack of confidence in their potential COVID-19 infection, without having undergone laboratory screening.

**Table 5:** Parent’s experience with vaccination and the COVID-19.

	Frequency (%)	95% confidence interval
Have you ever experienced any negative reactions from vaccinations?		
Yes	288 (19.49%)	13.07–23.18
No	921 (62.31%)	58.29–67.05
Not sure	269 (18.20%)	13.16–22.30
Have you received the seasonal influenza vaccination the previous year?		
Yes	566 (38.29%)	32.75–43.07
No	912 (61.71%)	56.04–65.17
Do not remember	–	–
Any of the following entities have been confirmed to have contracted the COVID-19 infection in a clinical setting?		
Me personally	271 (18.34%)	13.27–22.74
A member of my family	296 (20.03%)	17.18–24.38
One of my friends	143 (9.68%)	5.59–12.23
One of my co-workers one of my neighbors	36 (2.44%)	0.98–4.38
Nobody	109 (7.37%)	2.27–9.76
More than one laboratory examination	623 (42.15%)	39.84–46.19
Do you suspect that you may have had a COVID-19 infection or exposure without being tested?		
Yes	611 (41.34%)	36.54–46.17
No	867 (58.66%)	52.73–63.18
Not sure	–	–

## Discussion

Vaccination has emerged as a highly efficacious scientific methodology, providing substantial evidence of its ability to effectively control various contagious infections and contribute to the elimination of diseases. The current research endeavors to identify the factors that contribute to parents' inclination or hesitancy in vaccinating their children against the coronavirus. The parent's perspective of vaccination is one of the key elements in determining whether or not a child would receive a vaccine^30^. According to the World Health Organization (WHO) report (2020) on vaccination, there has been an upsurge of approximately 3.4 million unvaccinated children compared to the proportion during 2019 experienced a downfall in the overall proportion. Although investigations are being conducted to identify the causative factors of this reduction, there are specific myths that are being spread concerning several vaccinations^31^.

Although the COVID-19 vaccine is the most effective weapon to combat the ongoing epidemic, we observed a higher proportion of vaccine rejection and skepticism against the vaccine. According to a recent investigation in the United States (US), 21% of the proportion are quite sure of not receiving the vaccination, and about 4 out of 10 individuals would either definitively or possibly decline it. Multiple factors in different regions or community sectors are contributing to the rising percentage of vaccination rejection or reluctance^32,33^. To get an appropriate portrayal of parents' perceptions of the COVID-19 vaccination, we conducted an electronic survey that was specifically directed at those parents who willingly participated in the research investigation. The findings of our investigation are consistent with many prior studies. For instance, Mei-Xian Zhang et al. revealed that parental responses are stronger from females than from men and those female parents are more sensitive concerning the safety of offered vaccines. They also indicated that 46% of parents expressed their willingness to have their children immunized, however, the results of our study reported a relatively higher proportion of 61.77%^34^. A significant ratio of Pakistani parents with advanced educational backgrounds believed that vaccinations are not dangerous to their children. There is an expanding corpus of evidence that indicates that confidence in vaccination is also significant with high educational backgrounds^21,35,36^. In the present survey, nearly 34.83% of parents between the ages of 18 and 28 believe that vaccinations are safe for children, highlighting that younger Pakistani parents are more aware and knowledgeable than older groups and as a result know more about vaccinations and their benefits^34,37^. Considering the mortality rate among younger individuals was less than the elderly group, young people may have reduced perceptions of the risk of the pandemic, highlighting the significance of young parents accurately comprehending their essential role in the virus's propagation and family safety^21^.

Additionally, the findings of our study indicate that parents with two or fewer children exhibited a greater tendency towards receiving the coronavirus vaccination and harbored fewer concerns compared to the group with more than two children. The rising generation in Pakistan is more well-educated and well-informed than the elder generation here, which might be the explanation for why parents with fewer children are younger and more educated than parents with more children^37,38^. One of the underlying conditions for declining or delaying vaccination for young children could be multiparty^29,38^.

Parental concerns concerning vaccination safety and its negative consequences are widespread for a variety of reasons. Evidence has shown that the Rubella (MMR), Measles, and Mumps vaccinations and their connection to autism have also served an important part in vaccine reluctance and resistance previously which have raised concerns about both the COVID-19 and the MMR vaccination^39^. People have similar perceptions and fears concerning the COVID-19 vaccination owing to the unknown efficacy of the COVID-19 vaccine and some fallacies about vaccine-associated fertility problems^40,41^. The results of our survey are also in line with earlier research, which revealed that approximately 50% of parents were afraid that the COVID-19 vaccination would have an impact on their children's genes, fertility, or puberty^21,41^. According to the US surveillance statistics, children < 19 of age have developed multisystem inflammatory syndrome after receiving at least a single dose of the coronavirus vaccination^42^. Another piece of evidence was discovered that linked COVID-19 immunization to the development of multisystem inflammatory syndrome in 21 children^43^. The current study outcomes additionally highlighted that a significant fraction of the study participants was willing to get their children vaccinated against COVID-19 infection and also had a favorable attitude regarding the significance of doing so. According to a survey, almost 26% of parents repudiated getting their children vaccinated against COVID-19 infection due to concerns about negative consequences, however, the majority of them believed the opposite. Only 16% of participants in our research expressed the fear that the COVID-19 vaccination may impact their children's DNA. This is most likely due to a mix-up between messenger RNA vaccination and reverse genetics technology, which is frequently practiced for the genealogical modification of RNA pathogens from their complete cloned DNA and may potentially create viable countermeasures^44^. A considerable proportion of parents strongly believed that getting vaccinated may disrupt the puberty or fertility of their children. Based on the findings of Malik Sallam and colleagues*,* 23% of the populations in Kuwait and Jordan believe that immunizations might cause infertility^45^. Research findings on the long-term adverse consequences of the COVID-19 vaccination in humans are presently lacking in magnitude.

In our research, healthcare professionals (61.84%) and electronic media (38.16%) were the most trustworthy information sources for parents concerning vaccinations, which was found consistent with other earlier investigations that reported the same factors of reliance^46–48^. Contrarily, while electronic media are also excellent information sources, they are also linked to significantly larger misconceptions, misinformation, and skepticism about the COVID-19 vaccination. This is possibly related to how easily misleading information spreads across media platforms, particularly fake and inaccurate data about the efficacy of COVID-19 vaccinations^49^. In the Punjab province of Pakistan, a significantly higher proportion of parents (over 80%) encouraged rushing to undergo the COVID-19 vaccination, demonstrating that the study populace was concerned about its well-being. Additionally, there is a pressing concern for governments and public health organizations to advocate for comprehensive COVID-19 vaccination coverage, not only within Pakistan but also globally^49,50^.

The majority of study participants in our research concurred that vaccinations are commonly perceived as harmless and effective which corroborated many other prior studies. Another study reported that 20% of respondents' family members had COVID-19 infection, compared to 13% of respondents who worried their family members would become infected, which is less than the claimed rate of 34%^51^. Previous studies also examined many factors that contribute to parental vaccination reluctance and concluded that parents who had no plans to vaccinate their children against COVID-19 had much-increased levels of misinformation and skepticism about the COVID-19 vaccines. Additionally, they were less confident in the safety and effectiveness of the COVID-19 pediatric vaccination^52^.

Furthermore, 18.87% of parents expressed hesitation, believing that vaccinations are riskier for children than for adults. Almalki et al., also reported that the most reluctant parents to vaccinate their children were those who believed the vaccination offered minimal protection or who had reliability or effectiveness concerns. About 50% of the parents in our investigation did not believe that vaccines may pose a greater risk for children than adults. Comparable to this, another survey revealed that 59.8% of parents declared that they would be ready to vaccinate their children if it meant protecting them from COVID-19 and its consequences^53^. Other misconceptions about the COVID-19 vaccine for children have been identified, including the ones that the vaccine may trigger autism (15.2%), suspicion of pharmaceutical corporations (54.2%), and the virus produced by the governments themselves (48.6%)^54^. In a global assessment, approximately 37.7% voiced concern about the COVID-19 vaccine's health consequences, and 5.6% said that coronavirus is a self-limiting infection, negating the necessity for vaccination^55^. In a recent study of Egyptian students, over 22% of the participants suspected that vaccinations might have long-term genetic alteration consequences^56^. Globally, females are estimated to be more reluctant than males to have the COVID-19 vaccine^57^. Most probably, they are concerned about future fertility and puberty. Another research that included fifty states discovered that 37% of people believed vaccinations had influences only on female's health and 34% believed vaccines had influences only on male’s health^58,59^. According to a survey carried out in the USA, 41% of individuals suspected that the COVID-19 vaccination may disrupt their fertility or reproductive health, while 38% of participants were uncertain of the repercussions^60^. A relatively modest proportion of respondents to a nationwide survey, 2 according to Szilagyi et al., stated that they were inclined to have their children's COVID-19 vaccination^61^. In prior research of a Saudi community, a substantial ratio of parents intended to vaccinate their children against COVID-19^62^. It is particularly crucial to reduce the level of parental hesitation, fears, and other health considerations that are frequently reported. Additionally, according to some research, parents were less motivated to vaccinate themselves than their children. Children's vaccinations are decided by their parents. To boost COVID-19 vaccination acceptance in children, interventions are likely to emphasize primarily minimizing vaccine reluctance and enhancing parental uptake.

The emergence of the internet and electronic media platforms has substantially transformed the modern world by facilitating interpersonal connections and facilitating the widespread dispersion of information. Individuals can access a wide array of information regarding health-related matters on electronic media platforms. These platforms possess the capacity to furnish valuable information, yet simultaneously exhibit the propensity to disseminate inaccurate information, thereby engendering a sense of distrust and perplexity among users. To enhance parental acceptance of vaccines, health organizations, and ministries must disseminate precise information concerning vaccinations through electronic media channels. Decision-makers have to be cognizant of the importance of maintaining positive attitudes, as they play a pivotal role in fostering high levels of confidence. Furthermore, a comprehensive integration of multiple factors is essential to optimize the effective implementation of social marketing strategies within the realms of medicine and public health.

All the study participants in our investigation were Muslims and we anticipate that these potential insights may assuage parental reluctance regarding vaccination within other regions and countries with significant Muslim populations, where resistance to vaccinations exists due to religious beliefs. The outcomes of our research concerning the recognition of the significance of vaccinations are additionally promising because of the recent emergence of novel mutants. The findings of this study suggest that a significant proportion of the Pakistani population would be willing to accept the COVID-19 vaccination without hesitation, upon its availability and demonstrated efficacy. This initiative would not only contribute to saving human lives and containment of the virus but also ameliorate the socio-economic consequences posed by the pandemic on both individual citizens and nations.

### Limitations and future directions

Several factors might have influenced the specific research constraints. Due to the cross-sectional nature of the survey, the causal relationship between the variables under investigation remains uncertain. The findings may be subjected to potential overestimation or underestimation due to reliance on self-reported feedback. Secondly, following the prescribed criteria for sample size determination, the initial projected sample size of our study was 1537; however, after removing 59 because of inadequate and incomplete information, it was reduced to 1478. This fell short of the desired sample size and had to be adjusted by the collecting of more samples, which was unattainable owing to time and resource limitations. Thirdly, it should be noted that the survey was limited to a single city in the Punjab province, thus limiting the generalizability of the study outcomes to the diverse socio-economic groups residing in various provinces across Pakistan. Furthermore, it is plausible that individuals with restricted internet access could have been precluded from participating in the online questionnaire. The present investigation exclusively focused on the demographic of the literate population, thereby omitting those facing disabilities or social disadvantages as well as those lacking proficiency in English/Urdu language comprehension. We expect to have all of the necessary resources in the years ahead to conduct multicentric/national investigations. In addition, it is anticipated that future investigations will encompass individuals who possess limited English/Urdu comprehension by administering the survey in local languages as well.

## Conclusions

In conclusion, the outcomes of our study highlight potential perspectives on parents’ intents and attitudes toward vaccinating their children against COVID-19 infection. Initiatives to guarantee that children are vaccinated against coronavirus should be put into place and supported by healthcare professionals and legislators. They must also educate parents and family members about the significance of the COVID-19 vaccine. Healthcare awareness-supporting programs for educating parents should be designed and implemented. These insights might aid in the development of strategies to eradicate barriers in existing coronavirus vaccination programs and may vaccinate a larger children population to reduce the adverse consequences of the pandemic.

### Supplementary Information

Supplementary Information 1.

Supplementary Information 2.

## Data Availability

The data are available from the first author upon reasonable request.
