# Supplementary material for: Potential determinants of parental hesitancy to vaccinate their children against COVID-19 infection: a cross-sectional investigation
Source: Sci Rep. 2023 Dec 13;13:22161. doi: 10.1038/s41598-023-47863-6 (PMC10719250; doi:10.1038/s41598-023-47863-6)
Supplement: Supplementary file 1 — Supplementary Information 1. [file 41598_2023_47863_MOESM1_ESM.pdf]

STROBE Statement—Checklist of items that should be included in reports of ***cross-sectional studies***

**Title and abstract**		
	1	✓
**Introduction**		
Background/rationale	2	✓
Objectives	3	✓
**Methods**		
Study design	4	✓
Setting	5	✓
Participants	6	✓
Variables	7	✓
Data source/measurement	8	✓
Bias	9	✓
Study size	10	✓
Quantitative variables	11	✓
Statistical methods	12	✓
**Results**		
Participants	13	✓
Descriptive data	14	✓
Outcome data	15	✓
Main results	16	✓
**Discussion**		
Key results	17	✓
Limitations	18	✓
Interpretations	19	✓
**Other information**		
Funding	20	✓
