# Supplementary material for: Potential determinants of parental hesitancy to vaccinate their children against COVID-19 infection: a cross-sectional investigation
Source: Sci Rep. 2023 Dec 13;13:22161. doi: 10.1038/s41598-023-47863-6 (PMC10719250; doi:10.1038/s41598-023-47863-6)
Supplement: Supplementary file 2 — Supplementary Information 2. [file 41598_2023_47863_MOESM2_ESM.pdf]

Results of Pilot testing

Pilot testing was done with a sample of 35 participants.

- Descriptive statistics
- Reliability analysis
- Exploratory factor analysis (validity analysis)

Descriptive statistics

	Mean	SD	Min.	Max.	Skewness (<2)	Kurtosis (<7)
Q1	2.3	0.73	1	3	-1.21	-2.28
Q2	2.8	0.85	1	6	-1.43	1.96
Q3	2.9	0.43	1	6	-0.54	1.37
Q4	2.9	0.76	1	6	-1.01	- 0.76
Q5	3.5	0.51	1	6	-1.39	2.48
Q6	1.8	1.32	1	3	-0.88	0.56
Q7	2.7	0.77	1	3	-0.73	1.02
Q8	1.6	1.44	1	5	-1.54	- 3.12
Q9	1.7	1.65	1	3	-1.18	-2.76
Q10	2.4	0.87	1	3	-0.39	1.23
Q11	1.8	1.56	1	3	-0.64	- 1.65
Q12	5.4	2.95	1	6	-1.58	2.42
Q13	1.8	1.48	1	3	-1.49	2.19

Reliability analysis

The reliability of the item questionnaire has been checked using Cronbach's alpha for 35 samples for pilot testing, and the outcomes are presented below.

Items on the questionnaire	***α-Coeff.***
Have you been hesitant to vaccinate your child?	0.84
Do you believe that your child should receive the COVID-19 vaccine?	0.86
Are you willing to vaccinate your child against COVID-19 infection?	0.85
Do you avoid giving your child the COVID-19 vaccine because you are afraid of the adverse effects?	0.83
Do you have fears regarding the potential impact of the COVID-19 vaccine on the pubertal development or fertility of your child?	0.84
Do you think it's a good idea to get the COVID-19 vaccine as soon as possible?	0.87
Do you have fears that the COVID-19 vaccine could disrupt your child's DNA?	0.84
What level of satisfaction do you have with the services offered at the vaccination centers?	0.82
Have you ever experienced any negative reactions from vaccinations?	0.85
Have you received the seasonal influenza vaccination the previous year?	0.85
Any of the following entities have been confirmed to have contracted the COVID-19 infection in a clinical setting?	0.88
Do you suspect that you may have had a Coronavirus infection or exposure without being tested?	0.85

Exploratory factor analysis (EFA)

KMO and Bartlett's Test		
Kaiser-Meyer-Olkin Measure of Sampling Adequacy		0.923
Bartlett's Test of Sphericity	Approx. Chi-Square	234.19
	df	1477
	Sig.	0.000

Communalities

	Initial	Extraction
Q1	1.000	0.615
Q2	1.000	0.654
Q3	1.000	0.598
Q4	1.000	0.542
Q5	1.000	0.712
Q6	1.000	0.695
Q7	1.000	0.716
Q8	1.000	0.564
Q9	1.000	0.643
Q10	1.000	0.639
Q11	1.000	0.726
Q12	1.000	0.558
Q13	1.000	0.705

Extraction method: Principal component analysis

Total variance explained

Items	Initial eigen values			Extraction sums of squared loadings		
	Total	*% of variance*	*Cumulative %*	*Total*	*% of variance*	*Cumulative %*
1	4.881	37.55	37.55	4.881	37.55	37.55
2	1.714	13.18	50.73	1.714	13.18	50.73
3	0.995	7.65	58.39			
4	0.878	6.75	65.14			
5	0.797	6.13	71.27			
6	0.723	5.56	76.83			
7	0.698	5.37	82.20			
8	0.582	4.48	86.68			
9	0.469	3.61	90.29			
10	0.417	3.21	93.50			
11	0.354	2.72	96.22			
12	0.295	2.27	98.49			
13	0.197	1.52	100.00			
